# Effect of pre-ICU aspirin use on neuroinflammation and outcomes in patients with sepsis-associated encephalopathy

**DOI:** 10.3389/fneur.2026.1708039

**Published:** 2026-02-02

**Authors:** Zhenkun Xu, Qichao Yang, Hunian Li, Ting He

**Affiliations:** 1 Department of Emergency Medicine, Shiyan Renmin Hospital, Hubei, China; 2 Department of Neurology, Shiyan Renmin Hospital, Hubei, China

**Keywords:** aspirin, cerebral hemodynamics, neuroinflammation, prognosis, sepsis-related encephalopathy

## Abstract

**Objective:**

To investigate the effect of pre-ICU aspirin use on neuroinflammation and prognosis in sepsis-associated encephalopathy (SAE) patients.

**Methods:**

Clinical data of SAE patients admitted to our ICU (Mar 2022–Feb 2025) were retrospectively analyzed. Patients were grouped based on pre-admission aspirin use: exposed (*n* = 45) and non-exposed (*n* = 68). After 1:1 propensity score matching (age, infection source; caliper = 0.2), 42 matched pairs were compared. Cerebral hemodynamics (Vm, Vd, and Vs), coagulation function (PLT, TT, PT, and APTT), neuroinflammation markers (IL-6, TNF-α, and S100β), Glasgow Coma Scale (GCS), Sequential Organ Failure Assessment (SOFA) scores (admission, days 1, 3, and 5), ICU length of stay, adverse events, 28- and 60-day mortality were analyzed using appropriate statistical tests (t-test, χ^2^ test; *P* < 0.05 significant).

**Results:**

The exposed group had higher Vm, Vd, and Vs at all time points (*P* < 0.05). IL-6, TNF-α, and S100β levels were lower in the exposed group (*P* < 0.05). GCS scores were higher in the exposed group on days 3 and 5 (*P* < 0.05). Adverse event incidence, ICU stay, and 28-day mortality did not differ significantly (*P* < 0.05). The 60-day mortality was lower in the exposed group (*P* < 0.05).

**Conclusions:**

Pre-ICU aspirin use can improve cerebral hemodynamics, reduce neuroinflammation, and improve 60-day survival in SAE patients without increasing adverse reactions.

## 1 Introduction

Sepsis-associated encephalopathy (SAE) is a common complication in patients with sepsis. It is characterized by impaired consciousness and cognitive dysfunction, and it markedly increases both mortality and the risk of long-term neurological impairment (1). The pathogenesis of SAE is complex. It involves neuroinflammatory responses, disruption of the blood–brain barrier, cerebral microcirculatory dysfunction, and mitochondrial impairment. Among these mechanisms, excessive activation of neuroinflammation is considered a key factor leading to brain injury (2). Thus, reducing neuroinflammatory responses in patients with SAE has become a major focus in clinical treatment. However, there is currently no specific pharmacological therapy for SAE.

Recent studies have shown that sepsis can induce excessive platelet activation, which promotes cerebral microvascular thrombosis. Activated platelets also release a range of inflammatory mediators, thereby exacerbating neuroinflammation and impairing the integrity of the blood–brain barrier (3). In addition, accumulating evidence indicates that antiplatelet agents may have therapeutic effects on sepsis-related inflammation (4). Aspirin, a classic antiplatelet drug, has recently been reported to reduce systemic inflammation and improve microcirculation by inhibiting cyclooxygenase (COX) and the nuclear factor-κB pathway. Yet its impact on neuroinflammation and outcomes in patients with SAE remains unclear (5). Therefore, this study retrospectively analyzed the clinical data of patients with SAE to investigate whether regular aspirin use prior to ICU admission improves neuroinflammation and outcomes. The aim is to provide evidence for aspirin as an adjunctive therapy in SAE.

## 2 Materials and methods

### 2.1 General information

A retrospective analysis was conducted on the clinical data of patients with SAE admitted to the ICU of our Hospital from March 2022 to February 2025. Patients were divided into the exposure group (45 cases) and the non-exposure group (68 cases) according to aspirin use prior to admission. The study was approved by the Ethics Committee of our Hospital, and informed consent was waived.

Inclusion criteria were as follows: age between 45 and 75 years; meeting the diagnostic criteria for sepsis (6); diagnosis of SAE, defined as sepsis patients presenting with delirium, coma, other disturbances of consciousness, cognitive decline, or a Glasgow Coma Scale (GCS) score < 15, after excluding disturbances caused by the use of sedatives, analgesics, or anesthetic agents, other primary central nervous system disorders such as stroke, intracranial infection, or metabolic encephalopathy, and confirmed by laboratory tests and electroencephalography; ICU stay ≥48 h; clear medication history; uniform treatment protocol after admission; complete clinical records.

Exclusion criteria were: intracranial organic lesions or primary intracranial infections; long-term use (>7 d) of other antiplatelet or anticoagulant drugs before admission; congenital malformations or psychiatric disorders; severe chronic neurological diseases such as dementia, Parkinson's disease, or epilepsy; end-stage liver disease or renal failure; administration of high-dose glucocorticoids or immunosuppressants within 24 h before admission; active malignancy, metabolic disorders, or coagulopathy.

Given the retrospective and exploratory design of this study, no prospective sample size calculation was performed. All eligible patients admitted within the specified study period who met the inclusion and exclusion criteria were initially enrolled. To assess the adequacy of the resulting sample size for detecting a clinically meaningful difference in the primary outcome, a *post-hoc* power analysis was conducted following propensity score matching. With the final matched cohort of 42 patients per group, the study achieved a *post-hoc* power of 81% to detect the observed difference in 60-day mortality (effect size ϕ = 0.31) at a two-sided alpha level of 0.05.

### 2.2 Treatment

In the exposure group, patients had taken aspirin regularly within 30 days before admission (100 mg, National Medicine Standard HJ20160685, Bayer S.p.A.), 100 mg once daily. Patients in the non-exposure group had not taken aspirin or other antiplatelet agents within 30 days before admission. After admission, both groups received standard treatment, including infection control, vasoactive agents, fluid resuscitation, organ protection, and delirium prevention.

### 2.3 Observation indicators

1. *Cerebral hemodynamics:* at admission and on days 1, 3, and 5 after admission, transcranial Doppler (TCD, DWL Doppler-Box^®^, Germany) was used to monitor mean blood flow velocity (Vm), end-diastolic velocity (Vd), and peak systolic velocity (Vs) of the middle cerebral artery.
2. *Coagulation function:* at admission and on days 1, 3, and 5 after admission, 3 mL of cubital venous blood was collected and anticoagulated with 3.2% sodium citrate (1:9 ratio). Plasma was separated after centrifugation (3000 rpm, 10 cm, 15 min), and thrombin time (TT), prothrombin time (PT), and activated partial thromboplastin time (APTT) were measured using an automated coagulation analyser (Sysmex CS-2500^®^). An additional 2 mL of whole blood was collected for platelet count (PLT).
3. *Neuroinflammation-related markers:* at admission and on days 1, 3, and 5 after admission, 3 mL of cubital venous blood was collected. After resting for 30 min, the samples were centrifuged (2000 rpm, 12 cm, 10 min), and serum *was obtained. Levels of interleukin-6 (IL-6), tumor necrosis factor-*α (TNF-α), and S100β protein were determined using enzyme-linked immunosorbent assay (ELISA).
4. *GCS and SOFA scores:* at admission and on days 1, 3, and 5 after admission, the Glasgow Coma Scale (GCS) and Sequential Organ Failure Assessment (SOFA) were used to assess consciousness and organ function in both groups. GCS included eye, verbal, and motor responses, with a total score of 3–15. Scores of 3–8 indicated coma, 9–11 moderate impairment, 12–14 mild impairment, and 15 normal. SOFA covered six organ systems, with each system scored 0–4, yielding a total of 0–24. Higher scores indicated worse organ function.
5. *Adverse events:* adverse events during ICU treatment were recorded and compared between the two groups, including gastrointestinal bleeding, thrombocytopenia, and allergic reactions.
6. *Prognostic indicators:* ICU length of stay was recorded and compared. Mortality rates within 28 days and 60 days after admission were calculated (number of deaths/total cases × 100%).

### 2.4 Propensity score matching

Given the non-randomized, retrospective design of this study, a 1:1 propensity score matching (PSM) was performed to minimize potential confounding and improve the comparability of baseline characteristics between patients with and without pre-ICU aspirin exposure.

#### 2.4.1 Propensity score estimation and matching

The propensity score, defined as the conditional probability of receiving pre-ICU aspirin given the observed baseline covariates, was estimated for each patient using a multivariable logistic regression model. The dependent variable was pre-ICU aspirin use (yes/no). Covariates included in the model were selected based on clinical relevance and prior literature, encompassing: age, sex, Glasgow Coma Scale (GCS) score at ICU admission, primary source of infection (categorized as pulmonary, intra-abdominal, or urinary tract), and key comorbidities (hypertension, diabetes mellitus, and coronary artery disease). Patients in the aspirin-exposed group were then matched to those in the non-exposed group in a 1:1 ratio without replacement, using the nearest-neighbor matching algorithm within a caliper width set at 0.2 of the standard deviation of the logit of the propensity score.

#### 2.4.2 Balance diagnostics

**Figure 1:**
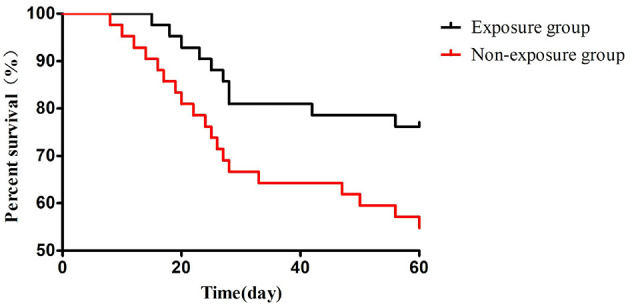
Kaplan-Meier curves comparing 60-day survival between patients with sepsis-associated encephalopathy who had pre-ICU aspirin exposure vs. those who did not. The Cox regression-derived hazard ratio for the aspirin-exposed group was 0.51 (95% CI: 0.27–0.97; *P* = 0.039).

The balance of baseline covariates between the two groups before and after matching was formally assessed using standardized mean differences (SMD). An SMD of less than or equal to 0.1 was considered indicative of adequate balance between the groups. The distributions of SMDs for all covariates are summarized in Table 1 and visualized in a Love plot in Figure 1. After matching, the SMD for each covariate was reduced to below 0.1, confirming that the matching procedure successfully achieved satisfactory balance. Consequently, 42 well-matched pairs (84 patients in total) were included in the final comparative analysis cohort.

**Table 1:** Comparison of clinical data between the two groups before matching [$\bar{x}$ ± s, n (%)].

**Variable**	**Exposure group (*n* = 45)**	**Non-exposure group (*n* = 68)**	***t*/χ^2^**	***P***
Sex			0.796	0.372
Male	27 (60.00)	35 (51.47)		
Female	18 (40.00)	33 (48.53)		
Age (years)	62.36 ± ±6.24	58.42 ± ±6.70	3.144	0.002
GCS score (points)	10.24 ± ±1.85	9.96 ± 1.78	0.806	0.422
Primary infection source			6.845	0.033
Pulmonary infection	25 (55.56)	21 (30.88)		
Intra-abdominal infection	12 (26.67)	29 (42.65)		
Urinary tract infection	8 (17.78)	18 (26.47)		
**Comorbidities**				
Hypertension	22 (48.89)	30 (44.12)	0.248	0.618
Diabetes mellitus	15 (33.33)	18 (26.47)	0.617	0.432
Coronary artery disease	9 (20.00)	11 (16.18)	0.272	0.602

### 2.5 Statistical methods

All statistical analyses were performed on the matched cohort using SPSS software (version 23.0; IBM Corp., Armonk, NY, USA). Continuous variables are presented as mean ± standard deviation (SD) if normally distributed, and were compared between the two groups using the independent samples t-test. For longitudinal data measured at multiple time points (e.g., cerebral hemodynamics, inflammatory markers, GCS, and SOFA scores), a two-way repeated-measures analysis of variance (ANOVA) was employed, with group and time as factors, followed by *post-hoc* tests for within-group and between-group comparisons at specific time points. Categorical variables are presented as frequencies and percentages, and were compared using the Chi-square test or Fisher's exact test, as appropriate. Survival analysis was conducted using the Kaplan-Meier method, and the survival curves of the two groups were compared using the log-rank test. A two-tailed *P* value of < 0.05 was considered statistically significant for all analyses.

## 3 Results

### 3.1 Comparison of clinical data before and after matching

Before matching, there were no significant differences between the two groups in sex, GCS score, or comorbidities (*P* > 0.05). However, significant differences were observed in age and primary infection source (*P* < 0.05). Age and primary infection source were therefore selected as key matching variables. A 1:1 nearest neighbor matching method was applied (caliper = 0.2), and 42 patients were finally included in each group. After matching, no significant differences were found between the two groups in sex, GCS score, comorbidities, age, or primary infection source (*P* > 0.05). See Tables 1 and 2.

**Table 2:** Comparison of clinical data between the two groups after matching [$\bar{x}$ ± s, n (%)].

**Variable**	**Exposure group (*n* = 42)**	**Non-exposure group (*n* = 42)**	***t*/χ^2^**	***P***
Sex			0.435	0.510
Male	25 (59.52)	22 (52.38)		
Female	17 (40.48)	20 (47.62)		
Age (years)	61.25 ± ±6.08	60.74 ± ±6.53	0.370	0.712
GCS score (points)	10.31 ± ±1.76	10.55 ± ±1.68	-−0.639	0.524
Primary infection source			0.193	0.908
Pulmonary infection	23 (54.76)	21 (50.00)		
Intra-abdominal infection	11 (26.19)	12 (28.57)		
Urinary tract infection	8 (19.05)	9 (21.43)		
**Comorbidities**				
Hypertension	20 (47.62)	18 (42.86)	0.192	0.661
Diabetes mellitus	14 (33.33)	12 (28.57)	0.223	0.637
Coronary artery disease	8 (19.05)	12 (28.57)	1.050	0.306

### 3.2 Comparison of cerebral hemodynamics at different time points between the two groups

At admission and on days 1, 3, and 5 after admission, Vm, Vd, and Vs showed a gradual increasing trend in both groups, with values in the Exposure group being higher than those in the Non-exposure group (*P* < 0.05). See Table 3.

**Table 3:** Comparison of cerebral hemodynamics between the two groups at different time points ($\bar{x}$ ± s, cm/s).

**Variable**	**Group**	**N**	**At admission**	**Day 1 after admission**	**Day 3 after admission**	**Day 5 after admission**
Vm	Exposure group	42	50.65 ± 3.24	54.32 ± 3.45^a^	60.18 ± 3.72^a^	66.35 ± 3.54^a^
	Non-exposure group	42	46.32 ± 2.98	48.76 ± 3.12^a^	55.43 ± 3.28^a^	61.25 ± 3.35^a^
	*t*		6.375	7.746	6.207	6.781
	*P*		<0.001	<0.001	<0.001	<0.001
Vd	Exposure group	42	29.87 ± 3.87	33.45 ± 3.65^a^	38.92 ± 3.24^a^	42.58 ± 3.05^a^
	Non-exposure group	42	25.38 ± 3.06	27.84 ± 3.18^a^	33.67 ± 2.87^a^	38.25 ± 2.54^a^
	*t*		5.898	7.510	7.861	7.070
	*P*		<0.001	<0.001	<0.001	<0.001
Vs	Exposure group	42	78.26 ± 8.45	82.15 ± 8.62^a^	88.93 ± 8.74^a^	94.54 ± 8.86^a^
	Non-exposure group	42	73.44 ± 7.96	76.98 ± 8.03^a^	84.25 ± 8.35^a^	90.38 ± 8.49^a^
	*t*		2.691	2.844	2.509	2.197
	*P*		0.009	0.006	0.014	0.031

Compared with admission, ^a^*P* < 0.05.

### 3.3 Comparison of coagulation function at different time points between the two groups

At admission and on days 1, 3, and 5 after admission, TT and PT gradually decreased, while PLT gradually increased in both groups (*P* < 0.05). No significant differences were observed between the two groups at any time point for TT, PT, APTT, or PLT (*P* > 0.05). See Table 4.

**Table 4:** Comparison of coagulation function between the two groups at different time points ($\bar{x}$ ± s).

**Variable**	**Group**	***N***	**At admission**	**Day 1 after admission**	**Day 3 after admission**	**Day 5 after admission**
TT(s)	Exposure group	42	28.33 ± 2.45	27.12 ± 2.30^a^	25.64 ± 1.98^a^	23.25 ± 1.32组隶属Variable^a^
	Non-exposure group	42	27.98 ± 2.19	26.85 ± 2.15^a^	25.30 ± 1.85^a^	22.87 ± 1.28^a^
	*t*		0.690	0.556	0.813	1.339
	*P*		0.492	0.580	0.418	0.184
PT(s)	Exposure group	42	15.26 ± 0.98	14.53 ± 0.87^a^	13.42 ± 0.79^a^	12.54 ± 0.73^a^
	Non-exposure group	42	15.53 ± 1.03	14.72 ± 0.91^a^	13.60 ± 0.82^a^	12.68 ± 0.75^a^
	*t*		−1.231	−0.978	−1.024	−0.867
	*P*		0.222	0.331	0.309	0.389
APTT(s)	Exposure group	42	34.05 ± 2.28	33.88 ± 2.15	34.12 ± 2.20	33.75 ± 2.10
	Non-exposure group	42	33.86 ± 2.03	33.72 ± 1.98	33.95 ± 2.05	33.60 ± 1.95
	*t*		0.403	0.355	0.366	0.339
	*P*		0.688	0.724	0.715	0.735
PLT ( × 10^9^/L)	Exposure group	42	87.45 ± 5.89	90.36 ± 5.54^a^	103.36 ± 6.02^a^	120.35 ± 11.05^a^
	Non-exposure group	42	89.12 ± 6.03	92.08 ± 5.89^a^	100.86 ± 6.24^a^	118.45 ± 10.38^a^
	*t*		−1.336	−1.436	1.945	0.844
	*P*		0.185	0.155	0.055	0.401

Compared with admission, ^a^*P* < 0.05.

### 3.4 Comparison of neuroinflammation-related markers at different time points between the two groups

At admission and on days 1, 3, and 5 after admission, IL-6, TNF-α, and S100β gradually decreased in both groups, with levels in the Exposure group being lower than those in the Non-exposure group (*P* < 0.05). See Table 5.

**Table 5:** Comparison of neuroinflammation-related markers between the two groups at different time points ($\bar{x}$ ± s).

**Variable**	**Group**	***N***	**At admission**	**Day 1 after admission**	**Day 3 after admission**	**Day 5 after admission**
IL-6 (mg/L)	Exposure group	42	586.49 ± 77.98	320.25 ± 50.34^a^	156.72 ± 30.18^a^	98.36 ± 20.35^a^
	Non-exposure group	42	674.26 ± 84.26	450.38 ± 60.12^a^	284.15 ± 45.67^a^	145.29 ± 34.26^a^
	*t*		−4.955	−10.755	−15.086	−7.633
	*P*		<0.001	<0.001	<0.001	<0.001
TNF-α (ng/L)	Exposure group	42	31.85 ± 4.72	24.16 ± 3.85^a^	17.54 ± 2.93	14.32 ± 2.45^a^
	Non-exposure group	42	36.48 ± 5.24	30.25 ± 4.56^a^	22.38 ± 3.64	18.45 ± 3.12^a^
	*t*		−4.255	−6.613	−6.713	−6.747
	*P*		<0.001	<0.001	<0.001	<0.001
S100β (μg/L)	Exposure group	42	0.48 ± 0.12	0.35 ± 0.09^a^	0.25 ± 0.07^a^	0.19 ± 0.05^a^
	Non-exposure group	42	0.63 ± 0.15	0.52 ± 0.11^a^	0.38 ± 0.09^a^	0.28 ± 0.07^a^
	*t*		−5.061	−7.752	−7.389	−6.780
	*P*		<0.001	<0.001	<0.001	<0.001

Compared with admission, ^a^*P* < 0.05.

### 3.5 Comparison of GCS and SOFA scores at different time points between the two groups

At admission and on days 1 after admission, no significant differences were observed in GCS or SOFA scores between the two groups (*P* > 0.05). On days 3 and 5, GCS scores in the Exposure group were higher than those in the Non-exposure group (*P* < 0.05). See Table 6.

**Table 6:** Comparison of GCS and SOFA scores between the two groups at different time points ($\bar{x}$ ± s, points).

**Variable**	**Group**	***N***	**At admission**	**Day 1 after admission**	**Day 3 after admission**	**Day 5 after admission**
GCS	Exposure group	42	10.31 ± 1.76	10.03 ± 1.58	11.25 ± 0.84^a^	12.08 ± 0.93^a^
	Non-exposure group	42	10.55 ± 1.68	10.22 ± 1.53	11.89 ± 0.76^a^	12.53 ± 0.84^a^
	*t*		−0.639	−0.560	−3.661	2.327
	*P*		0.524	0.577	<0.001	0.022
SOFA	Exposure group	42	11.54 ± 2.86	10.22 ± 2.15^a^	9.08 ± 1.84^a^	5.95 ± 1.32^a^
	Non-exposure group	42	11.82 ± 3.02	10.45 ± 2.64^a^	9.56 ± 1.93^a^	6.38 ± 1.75^a^
	*t*		−0.436	−0.438	−1.167	−1.271
	*P*		0.664	0.663	0.247	0.207

Compared with admission, ^a^*P* < 0.05.

### 3.6 Comparison of adverse events between the two groups

The incidence of adverse events in the Exposure group was not significantly different from that in the Non-exposure group (*P* > 0.05). See Table 7. A detailed assessment using the Common Terminology Criteria for Adverse Events (CTCAE) version 5.0 revealed that all reported events were mild to moderate in severity (Grade 1 or 2). Specifically, gastrointestinal bleeding events were Grade 1 (mild), thrombocytopenia was Grade 2 (moderate), and allergic reactions were Grade 1 (mild). None of the events were severe (Grade ≥3), required surgical intervention, blood transfusion, or led to discontinuation of care.

**Table 7:** Comparison of adverse events between the two groups, n (%).

**Group**	***N***	**Gastrointestinal bleeding**	**Thrombocytopenia**	**Allergic reaction**	**Total incidence**
Exposure group	42	2 (4.76)	2 (4.76)	1 (2.38)	5 (11.90)
Non-exposure group	42	1 (2.38)	0 (0.00)	1 (2.38)	2 (4.76)
χ^2^					0.623
*P*					0.430

### 3.7 Comparison of prognostic indicators between the two groups

ICU length of stay and 28-day mortality in the Exposure group were not significantly different from those in the Non-exposure group (*P* > 0.05). The 60-day mortality in the Exposure group was lower than that in the Non-exposure group (*P* < 0.05). See Table 8. To further visualize this survival benefit, we performed a Kaplan-Meier survival analysis. As shown in Figure 1, the survival probability over the 60-day period was consistently higher in the Exposure group compared to the Non-exposure group. A Cox proportional hazards regression model, applied after confirming that the proportional hazards assumption was not violated (Schoenfeld residual test, *P* > 0.05), quantified this benefit. Pre-ICU aspirin use was associated with a significantly reduced risk of 60-day mortality (Hazard Ratio = 0.51, 95% Confidence Interval: 0.27 to −0.97, *P* = 0.039).

**Table 8:** Comparison of prognostic indicators between the two groups [$\bar{x}$ ± s, n (%)].

**Group**	***N***	**ICU length of stay (days)**	**28-day mortality**	**60-day mortality**
Exposure group	42	8.75 ± 2.01	8 (19.05)	10 (23.81)
Non-exposure group	42	9.38 ± 2.05	14 (33.33)	19 (45.24)
*t*/χ^2^		−1.422	2.217	4.266
*P*		0.159	0.136	0.039

## 4 Discussion

SAE is a diffuse brain dysfunction caused by sepsis. It is a secondary encephalopathy without evidence of direct central nervous system infection or structural brain injury. Its incidence in patients with sepsis is as high as 50%−70% (7, 8). With improvements in medical care, the mortality rate of sepsis has declined. However, the pathogenesis of SAE remains complex, the diagnostic rate is still low, and effective strategies to protect the nervous system are lacking. As a result, many survivors continue to suffer from neurological disorders such as cognitive impairment and memory decline, which severely affect their quality of life (9). Therefore, exploring effective drugs for the prevention and treatment of SAE is of great significance in improving patient outcomes.

Coagulation activation and inflammatory response are the two main host defense pathways in sepsis (10). At the early stage of infection, endothelial cell injury activates both coagulation and inflammation, leading to platelet activation and microthrombus formation. This mechanism helps to limit pathogen spread. As the disease progresses, however, excessive platelet activation releases inflammatory mediators and procoagulant substances, causing imbalance between pro-inflammatory and anti-inflammatory responses and disruption of the coagulation–fibrinolysis system. This triggers a systemic inflammatory storm, microcirculatory disturbance, and disseminated intravascular coagulation, eventually resulting in multiple organ dysfunction. In the development of SAE, uncontrolled interactions between inflammation and coagulation disrupt the blood–brain barrier, promote inflammatory cell infiltration and cerebral microthrombosis, and ultimately lead to neuroinflammation and brain dysfunction (11). Thus, inhibition of excessive platelet activation may be an important intervention to improve SAE outcomes.

Aspirin is a commonly used antiplatelet drug. It exerts its antiplatelet effect by irreversibly inhibiting COX-1 activity and reducing the production of thromboxane A2 (TXA2). A nationwide cohort study reported (12) that, compared with septic patients who did not receive any antiplatelet therapy prior to admission, those who used aspirin had significantly lower 90-day mortality and longer mean survival. Lavie et al. (13) also found that sepsis patients with long-term aspirin use had higher survival rates than non-users. Subgroup analysis indicated greater benefit among patients with chronic obstructive pulmonary disease or those on long-term β-blocker therapy. These findings suggest that aspirin confers benefits to patients with sepsis. It is therefore hypothesized that aspirin may also improve outcomes in SAE.

In this study, a retrospective design was used to explore the effect of pre-admission aspirin use on SAE patients. Given the multiple confounding factors inherent to retrospective studies, propensity score matching was applied to minimize bias, allowing a more accurate assessment of aspirin's impact on outcomes. The results showed that the 60-day mortality in the aspirin exposure group was significantly lower than in the non-exposure group, consistent with the above studies. This confirms that regular aspirin use within 30 days before admission helps reduce mortality. However, Rabouel et al. (14) reported that platelet P2Y12 receptor antagonists may not be beneficial in patients with sepsis or septic shock. Sullerot et al. (15) also found no significant association between aspirin use and reduced mortality in elderly patients with severe pneumonia. Such discrepancies may be related to differences in study populations, treatment doses, and treatment duration, with the latter two likely being more important.

An important observation in our study is that patients in the aspirin exposure group already exhibited significantly lower levels of neuroinflammation markers (IL-6, TNF-α, S100β) at the time of ICU admission compared to the non-exposure group. This baseline difference, as rightly noted, could be a potential confounder affecting the subsequent prognosis. We postulate that this difference is not merely coincidental but may, in fact, reflect the cumulative anti-inflammatory and neuroprotective effects of pre-admission aspirin use. By mitigating the systemic inflammatory response and platelet-mediated neuroinflammation in the early or pre-clinical stages of sepsis, chronic aspirin therapy might have modulated the initial severity of SAE. Therefore, the improved cerebral hemodynamics, lower neuroinflammation during ICU stay, and reduced 60-day mortality in the exposure group could be interpreted as the continuation of this pre-established protective effect, rather than solely an acute intervention after ICU admission. While propensity score matching balanced age and infection source, unmeasured confounding due to the inherent severity of the initial septic insult cannot be entirely ruled out.

S100β is a neuro-specific protein primarily secreted by astrocytes. It promotes neuronal growth, repair, and supports neuronal nutrition. Under normal conditions, the levels of S100β in serum and cerebrospinal fluid are very low. However, neuronal injury induces astrocyte apoptosis, triggering substantial synthesis and release of S100β, which leads to a marked increase in circulating S100β levels (16, 17). Therefore, S100β can serve as a sensitive biomarker for neurological injury. Song et al. (18) reported that serum S100β was significantly higher in SAE patients than in non-SAE patients, and it was closely associated with patient outcomes.

IL-6 and TNF-α are common inflammatory cytokines that can activate microglia and promote S100β release. Elevated S100β can, in turn, stimulate astrocytes to produce more IL-6 and TNF-α, forming a positive feedback loop that exacerbates neuroinflammation (19). Transcranial color Doppler ultrasonography is a non-invasive method for assessing cerebral function. Tong et al. (20) suggested that combining Vm, Vd, and Vs measurements with serum biomarkers can improve the diagnostic efficiency for SAE. Previous studies have mainly focused on the effect of aspirin on survival outcomes in sepsis, without adequately considering its impact on neuroinflammation and cerebral blood flow.

In this study, IL-6, TNF-α, and S100β levels in the aspirin exposure group were lower than those in the non-exposure group at admission and on days 1, 3, and 5 after admission. Vm, Vd, and Vs were higher in the aspirin group than in the non-exposure group. These findings suggest that pre-admission aspirin use may reduce neuroinflammation and improve cerebral blood flow, thereby helping to control disease progression. Mechanistically, aspirin exerts neuroprotective effects by inhibiting cyclooxygenase-mediated prostaglandin synthesis, reducing pro-inflammatory cytokine levels in the central nervous system (21). In addition, Wang et al. (22) reported that low-dose aspirin possesses both anti-inflammatory properties and promotes inflammation resolution via aspirin-triggered lipoxins, which mitigates vasogenic edema caused by blood–brain barrier disruption and helps regulate cerebral blood flow.

During the pathological process of sepsis, an imbalance in systemic inflammatory responses can lead to thrombocytopenia. The underlying mechanisms involve abnormal platelet aggregation, suppressed platelet production, and excessive consumption (23). When using antiplatelet agents clinically, careful consideration of drug selection and dosage is required to avoid excessive inhibition of platelet quantity or function, which could increase the risk of gastrointestinal or systemic bleeding. Some studies have confirmed that aspirin exerts antithrombotic effects by modulating the coagulation cascade (24). Therefore, close monitoring of coagulation function and platelet count is necessary during antiplatelet therapy.

Gong et al. (25) reported that aspirin use in SAE patients was associated with improved short-term and long-term survival, without significantly increasing the risk of gastrointestinal bleeding or thrombocytopenia. Low-dose aspirin (81 mg/day) may provide better long-term survival benefits than high-dose aspirin (325 mg/day). In this study, patients who regularly used 100 mg/day of aspirin were selected. The results showed that TT and PT gradually decreased, PLT gradually increased, and APTT remained stable over time in both groups. This indicates that coagulation function and platelet count improved with effective treatment. Inter-group comparison revealed no significant differences in TT, PT, APTT, or PLT, and complications such as thrombocytopenia or gastrointestinal bleeding were also similar. These findings suggest that reasonable aspirin use does not exacerbate coagulation dysfunction or thrombocytopenia in SAE patients.

Furthermore, with prolonged treatment, GCS and SOFA scores improved in both groups, with the aspirin exposure group showing more pronounced improvement in GCS scores. No significant difference was observed in SOFA scores between the groups. Wu et al. (26) reported that aspirin combined with standard sepsis therapy could reduce SOFA scores compared with standard therapy alone, which differs from the present findings. This discrepancy may be due to differences in study populations. SOFA scores are an important tool for assessing organ dysfunction in sepsis, but in SAE patients, neurological damage is more severe than in general sepsis. Thus, the effect of aspirin on SOFA scores may be masked by the severity of neurological injury. Additionally, persistent consciousness impairment in SAE patients affects the neurological component of SOFA, which only accounts for a portion of the overall score, resulting in no significant change in total SOFA scores.

This study has several limitations that should be considered when interpreting the results. First, the retrospective and single-center design, despite the use of propensity score matching, carries an inherent risk of residual confounding from unmeasured variables. The findings from our single institution with a relatively small matched sample size (42 pairs) may limit the generalizability of the results to broader or different populations. Second, the definition of pre-ICU aspirin use relied on documented medication history. We lacked precise data on the specific indication for aspirin therapy, patient adherence, and the exact duration of use prior to admission. These factors could influence both the accuracy of exposure classification and the observed treatment effects. Third, our follow-up was limited to 60 days, and the impact of pre-ICU aspirin use on long-term survival remains unknown, which is crucial for fully assessing the clinical value of the intervention. Finally, as a retrospective study, no prospective sample size calculation was performed, although a *post-hoc* analysis indicated adequate power for the primary mortality outcome.

## 5 Conclusion

In this retrospective observational study, pre-ICU aspirin use was associated with improved cerebral hemodynamics, reduced neuroinflammation, and a lower 60-day mortality in patients with sepsis-associated encephalopathy, without increasing adverse reactions. These findings suggest a potential benefit of prior aspirin use, but do not establish a causal therapeutic effect. Further multicenter, prospective, and ideally randomized studies are warranted to confirm these observations and clarify the role of aspirin in the management of SAE.

## Data Availability

The original contributions presented in the study are included in the article/supplementary material, further inquiries can be directed to the corresponding authors.

## References

[1] Zhou H Chang DH Kang YD. Research progress of high-mobility group box 1 protein in sepsis and sepsis-associated acute kidney injury. Clin Misdiagn Misther. (2023) 36:138–42. doi: 10.3969/j.issn.1002-3429.2023.01.030

[2] Li J Jia Q Yang L Wu Y Peng Y Du L . Sepsis-associated encephalopathy: mechanisms, diagnosis, and treatments update. Int J Biol Sci. (2025) 21:3214–28. doi: 10.7150/ijbs.102234 40384873 PMC12080397

[3] Jiang H Chen S Gui X Li Y Sun Y Zhu H . Platelet NLRP6 protects against microvascular thrombosis in sepsis. Blood (2025) 146:382–95. doi: 10.1182/blood.2025028739 40373277

[4] Kim SH Kim KH. Effects of prior antiplatelet and/or nonsteroidal anti-inflammatory drug use on mortality in patients undergoing abdominal surgery for abdominal sepsis. Surgery (2023) 174:611–7. doi: 10.1016/j.surg.2023.05.020 37385867

[5] Jiang S Xu J Ke C Huang P. Impact of P2Y12 inhibitors on clinical outcomes in sepsis-3 patients receiving aspirin: a propensity score matched analysis. BMC Infect Dis. (2024) 24:575. doi: 10.1186/s12879-024-09421-x 38862910 PMC11167871

[6] Emergency Emergency Physician Branch of Chinese Medical Doctor Association Shock Shock and Sepsis Committee of Chinese Research Hospital Association. Chinese Guidelines for Emergency Treatment of Sepsis/Septic Shock. J Clin Emerg. (2018) 19:567–88. doi: 10.13201/j.issn.1009-5918.2018.09.001

[7] Abudeke SL Xiao D Lv XW Guo RN Lu W. Advances in early diagnosis and treatment of sepsis-associated encephalopathy. China Med Sci. (2023) 13:75–8. doi: 10.3969/j.issn.2095-0616.2023.08.021

[8] Pan S Lv Z Wang R Shu H Yuan S Yu Y . Sepsis-induced brain dysfunction: pathogenesis, diagnosis, and treatment. Oxid Med Cell Longev. (2022) 2022:1328729. doi: 10.1155/2022/1328729 36062193 PMC9433216

[9] Huang Y Chen R Jiang L Li S Xue Y. Basic research and clinical progress of sepsis-associated encephalopathy. J Intensive Med. (2021) 1:90–5. doi: 10.1016/j.jointm.2021.08.002 36788800 PMC9923961

[10] Jacobi J. The pathophysiology of sepsis-2021 update: part 1, immunology and coagulopathy leading to endothelial injury. Am J Health Syst Pharm. (2022) 79:329–37. doi: 10.1093/ajhp/zxab380 34605875 PMC8500113

[11] Iba T Umemura Y Wada H Levy JH. Roles of coagulation abnormalities and microthrombosis in sepsis: pathophysiology, diagnosis, and treatment. Arch Med Res. (2021) 52:788–97. doi: 10.1016/j.arcmed.2021.07.003 34344558

[12] Hsu WT Porta L Chang IJ Dao QL Tehrani BM Hsu TC . Association between aspirin use and sepsis outcomes: a national cohort study. Anesth Analg. (2022) 135:110–7. doi: 10.1213/ANE.0000000000005943 35245223

[13] Lavie I Lavie M Gafter-Gvili A Halperin E Abramovich-Yoffe H Avni T. Chronic aspirin use and survival following sepsis-A propensity-matched, observational cohort study. Clin Microbiol Infect. (2022) 28:1287.e1–1287.e7. doi: 10.1016/j.cmi.2022.04.010 35533971

[14] Rabouel Y Magnenat S Delabranche X Gachet C Hechler B. Platelet P2Y12 receptor deletion or pharmacological inhibition does not protect mice from sepsis or septic shock. TH Open. (2021) 5:e343–52. doi: 10.1055/s-0041-1733857 34447900 PMC8384481

[15] Sullerot C Bouiller K Laborde C Gilis M Fèvre A Hacquin A . Premorbid aspirin use is not associated with lower mortality in older inpatients with SARS-CoV-2 pneumonia. Geroscience (2022) 44:573–83. doi: 10.1007/s11357-021-00499-8 34993763 PMC8736303

[16] Honore PM Redant S Kaefer K Barreto Gutierrez L Kugener L Attou R . Higher levels of S-100β-a biomarker of astrocyte and glial activation were associated with a greater delirium duration in sepsis and traumatic brain injury patients: beware of some confounders!. Crit Care Med. (2021) 49:e736–7. doi: 10.1097/CCM.0000000000004990 34135292

[17] Zhang N Xie K Yang F Wang Y Yang X Zhao L. Combining biomarkers of BNIP3 L, S100B, NSE, and accessible measures to predict sepsis-associated encephalopathy:a prospective observational study. Curr Med Res Opin. (2024) 40:575–82. doi: 10.1080/03007995.2024.2322059 38385550

[18] Song Q Li YG. The prognostic value of serum central nervous system-specific protein and high mobility group box-1 protein in children with sepsis-associated encephalopathy. Chin J Clinicians. (2023) 51:1237–40. doi: 10.3969/j.issn.2095-8552.2023.10.033

[19] Deng JC Wang D Wang M Li H. Protective effect and mechanism of xingnao enema on brain injury in septic mice. Chin J Emerg Tradit Med. (2024) 33:1542–6. doi: 10.3969/j.issn.1004-745X.2024.09.008.

[20] Tong X Liu SR Wang W. Value of transcranial colour doppler combined with serum NSE, EAA, and Occludin in early diagnosis of sepsis-associated encephalopathy. J Cent South Med Sci. (2025) 53:137–40. doi: 10.15972/j.cnki.43-1509/r.2025.01.034

[21] Eisen DP Leder K Woods RL Lockery JE McGuinness SL Wolfe R . Effect of aspirin on deaths associated with sepsis in healthy older people (ANTISEPSIS): a randomised, double-blind, placebo-controlled primary prevention trial. Lancet Respir Med. (2021) 9:186–95. doi: 10.1016/S2213-2600(20)30411-2 32950072 PMC7957956

[22] Wang L Li B Zuo L Pei F Nie Y Liu Y . Aspirin therapy and 28-day mortality in ICU patients: a retrospective observational study from two large databases. Clin Ther. (2023) 45:316–332. doi: 10.1016/j.clinthera.2023.02.005 36973090

[23] Xu X Wang Y Tao Y Dang W Yang B Li Y. The role of platelets in sepsis: a review. Biomol Biomed. (2024) 24:741–52. doi: 10.17305/bb.2023.10135 38236204 PMC11293227

[24] Almeida TML Freitas FGR Figueiredo RC Houly SG Azevedo LCP Cavalcanti AB . Acetylsalicylic acid treatment in patients with sepsis and septic shock: a phase 2, placebo-controlled, randomized clinical trial. Crit Care Med. (2025) 53:e269–281. doi: 10.1097/CCM.0000000000006564 39982179

[25] Gong QQ Yuan ZN She TY Ge F Zhang Y. Aspirin improves short and long term survival outcomes of patients with sepsis associated encephalopathy. Sci Rep. (2025) 15:222–42. doi: 10.1038/s41598-025-08075-2 40595183 PMC12215694

[26] Wu SL Niu KX Xu Y Liu C Deng XM. Effect of antiplatelet therapy on sepsis-induced inflammatory response, platelet function, and therapeutic outcomes. Chin J Gen Pract. (2022) 20:1311–4. doi: 10.16766/j.cnki.issn.1674-4152.002584

